# Revealing the full-length transcriptome of caucasian clover rhizome development

**DOI:** 10.1186/s12870-020-02637-4

**Published:** 2020-09-16

**Authors:** Xiujie Yin, Kun Yi, Yihang Zhao, Yao Hu, Xu Li, Taotao He, Jiaxue Liu, Guowen Cui

**Affiliations:** College of Animal Science and Technology, Northeast Agricultural University, No.600 Changjiang Street, Xiangfang District, Harbin, 150030 Heilongjiang China

**Keywords:** Caucasian clover (*Trifolium ambiguum* M. Bieb.), Full-length transcriptome, RNA-seq, Rhizome, Plant hormone, TFs

## Abstract

**Background:**

Caucasian clover (*Trifolium ambiguum* M. Bieb.) is a strongly rhizomatous, low-crowned perennial leguminous and ground-covering grass. The species may be used as an ornamental plant and is resistant to cold, arid temperatures and grazing due to a well-developed underground rhizome system and a strong clonal reproduction capacity. However, the posttranscriptional mechanism of the development of the rhizome system in caucasian clover has not been comprehensively studied. Additionally, a reference genome for this species has not yet been published, which limits further exploration of many important biological processes in this plant.

**Result:**

We adopted PacBio sequencing and Illumina sequencing to identify differentially expressed genes (DEGs) in five tissues, including taproot (T1), horizontal rhizome (T2), swelling of taproot (T3), rhizome bud (T4) and rhizome bud tip (T5) tissues, in the caucasian clover rhizome. In total, we obtained 19.82 GB clean data and 80,654 nonredundant transcripts were analysed. Additionally, we identified 78,209 open reading frames (ORFs), 65,227 coding sequences (CDSs), 58,276 simple sequence repeats (SSRs), 6821 alternative splicing (AS) events, 2429 long noncoding RNAs (lncRNAs) and 4501 putative transcription factors (TFs) from 64 different families. Compared with other tissues, T5 exhibited more DEGs, and co-upregulated genes in T5 are mainly annotated as involved in phenylpropanoid biosynthesis. We also identified betaine aldehyde dehydrogenase (*BADH*) as a highly expressed gene-specific to T5. A weighted gene co-expression network analysis (WGCNA) of transcription factors and physiological indicators were combined to reveal 11 hub genes (MEgreen-GA3), three of which belong to the HB-KNOX family, that are up-regulated in T3. We analysed 276 DEGs involved in hormone signalling and transduction, and the largest number of genes are associated with the auxin (IAA) signalling pathway, with significant up-regulation in T2 and T5.

**Conclusions:**

This study contributes to our understanding of gene expression across five different tissues and provides preliminary insight into rhizome growth and development in caucasian clover.

## Background

Caucasian clover (*Trifolium ambiguum* M. Bieb.), also known as Kura clover, is a low-crowned, strongly rhizomatous perennial legume [1]; the species originates from the region encompassing Caucasian Russia, eastern Turkey and northern Iran [2]. Caucasian clover can protect lawns as a flowering species and an ornamental plant [3]. Compared with white clover, caucasian clover has lower fiber concentrations, greater protein concentrations and forage digestibility [4] outperforming in high-aluminum soils [5] and providing high-quality fodder during the year when white clover growth is poor [6]. Caucasian clover has deep, semi-woody, usually branched main roots, and many branched roots grow new plantlets, either at the ends or nodes [7–9]. The species can tolerate continuous grazing by cattle (*Bos taurus*) [7], extreme winter temperatures [8], seasonal moisture deficit and many serious diseases that affect other types of clover [9, 10]. These features are attributed to its prominent primary roots, low-spread crowns and well-developed rhizome systems [11].

In plants without reference genomes, high-throughput RNA sequencing (RNA-seq) technology has become a fast and effective way to master the molecular mechanisms of plants for research purposes [12–14]. This technology has been applied to the study of various rhizomatous species such as sorghum (*Sorghum halepense* and *Sorghum propinquum*) [15, 16], bamboo (*Phyllostachys praecox*) [17], *Oryza longistaminata* [18–20], *Equisetum hyemale* [21], *Panax ginseng* [22], *Phragmites australis* [23], tropical lotus (*Nelumbo nucifera*) [24], CangZhu (*Atractylodes lacea*) [25], *Ligusticum chuanxiong* [26], ginger (*Zingiber officinale*) [27], and *Miscanthus lutarioriparius* [12]. Studies have shown that plant rhizomes are rich in growth regulators, which are related to the energy, metabolism, and hormones pathways of rhizome development. For example, 48 important transcription factors (TFs) belonging to the bHLH, YABBY, NAM, TCP, TALE, and AP2 families are expressed specifically or in abundance in the shoot tip and elongation regions of *Oryza longistaminata* [18–20].

In recent years, an increasing number of full-length transcriptomes have been generated by Pacific Biosciences (PacBio) sequencing. PacBio sequencing is a single-molecule sequencing technology with a longer read length than second-generation sequencing and an average read length of up to 15 KB, and is also called Single-molecule, real-time (SMRT). This technology not require assembly and can completely retain the entire sequence from the 3′ to 5′ ends of an RNA, but with a higher error rate; moreover, the second-generation stepwise approach can correct mistakes [28–31]. PacBio has been utilized to detect more than 42,280 different splicing isoforms and a large number of alternative splicing (AS) events were found to be associated with the rhizome system and assist genome annotation in orchardgrass [32, 33]. The results indicate that posttranscriptional regulation plays an important role in the rhizome system. Moreover, a combination of Illumina and PacBio sequencing applied to various root tissues, particularly the periderm, has provided a more complete view of the Danshen *(Salvia miltiorrhiza)* transcriptome [34]. PacBio sequencing and RNA-seq analysis together have also been used to identify differentially expressed transcripts along a developmental gradient from the shoot apex to the fifth internode of *Populus P. deltoides×P. euramericana cv*.*‘Nanlin895’*, showing 15,838 differentially expressed transcripts, 1216 of which are TFs [35].

Compared with traditional herbage legumes, such as white clover and red clover, the rhizome is one of the most distinctive characteristics of caucasian clover [9, 36]. The rhizome system has important functions in energy storage, transport and vegetative reproduction [37–40]. We combined Illumina and PacBio sequencing to generate a complete full-length transcriptome of caucasian clover by analysing gene expression in five different tissues and identifying genes related to rhizome development in caucasian clover rhizomes. The obtained results are an excellent reference for further functional characterization to elucidate their roles in rhizome differentiation, growth and development.

## Results

### Analysis of PacBio sequencing datasets

Transcriptome sequencing of the caucasian clover rhizome was completed, and 19.82 GB of clean data were obtained using one cell. We identified 658,323 reads of inserts (ROIs) with a mean length of 2286 bp, a quality of 0.94, 12 passes from 720,832 polymerase reads with full passes> = 0 and a predicted consensus accuracy> 0.8 (Table 1). In total, the ROIs included 62.87% (449,460) full-length (FL) reads and 29.4% (193,513) non full-length reads of the entire transcriptome sequence from the 5′ to the 3′ end and polyA tail. Additionally, the number of full-length, non-chimeric (FLNC) reads was 441,885, with an average FLNC reads length of 1969 bp (Table 1). The main number distributions of cDNA and ROIs are shown in Additional file 1: Figure S1a and S1b.

**Table 1:** Statistics of the PacBio sequencing data, ROI databases and ICE clustering

cDNA library	1-6 KB
PacBio sequencing	
polymerase reads	720,832
Reads of insert	658,323
Read bases of insert	1,505,553,284
Mean read length of insert	2286
Mean read quality of insert	0.94
Mean number of passes	12
Date size (GB)	19.82
ROI databases	
Number of five prime reads	506,205
Number of three prime reads	521,059
Number of poly-A reads	514,174
Number of filtered short reads	15,350
Number of non-full-length reads	193,513
Number of full-length reads	449,460
Number of full-length non-chimeric reads	441,885
Average full-length non-chimeric read length	1969
ICE clustering	
Number of consensus isoforms	227,516
Average consensus isoforms read length	2086
Number of polished high-quality isoforms	148,836
Number of polished low-quality isoforms	78,366
Percent of polished high-quality isoforms (%)	65,42
Non-redundant transcripts	80,654

KB (kilobase): A commonly used length unit for DNA, indicating one thousand base pairs. GB: A unit used to measure the amount of data, where 1 GB = 1,000,000 bp.

As PacBio sequencing results have a high error rate, FLNC reads were clustered using the iterative clustering for error correction (ICE) algorithm and corrected with the Illumina Hiseq2500 platform to correct errors. We generated 227,516 consensus isoforms with an average consensus isoform length of 2086 bp, including 148,836 high-quality isoforms (Table 1). We successfully obtained 80,654 non-redundant transcripts using CD-HIT [41] for caucasian clover rhizomes analysis.

### Prediction of ORFs, SSRs and lncRNAs and identification of AS events

**Fig. 1:**
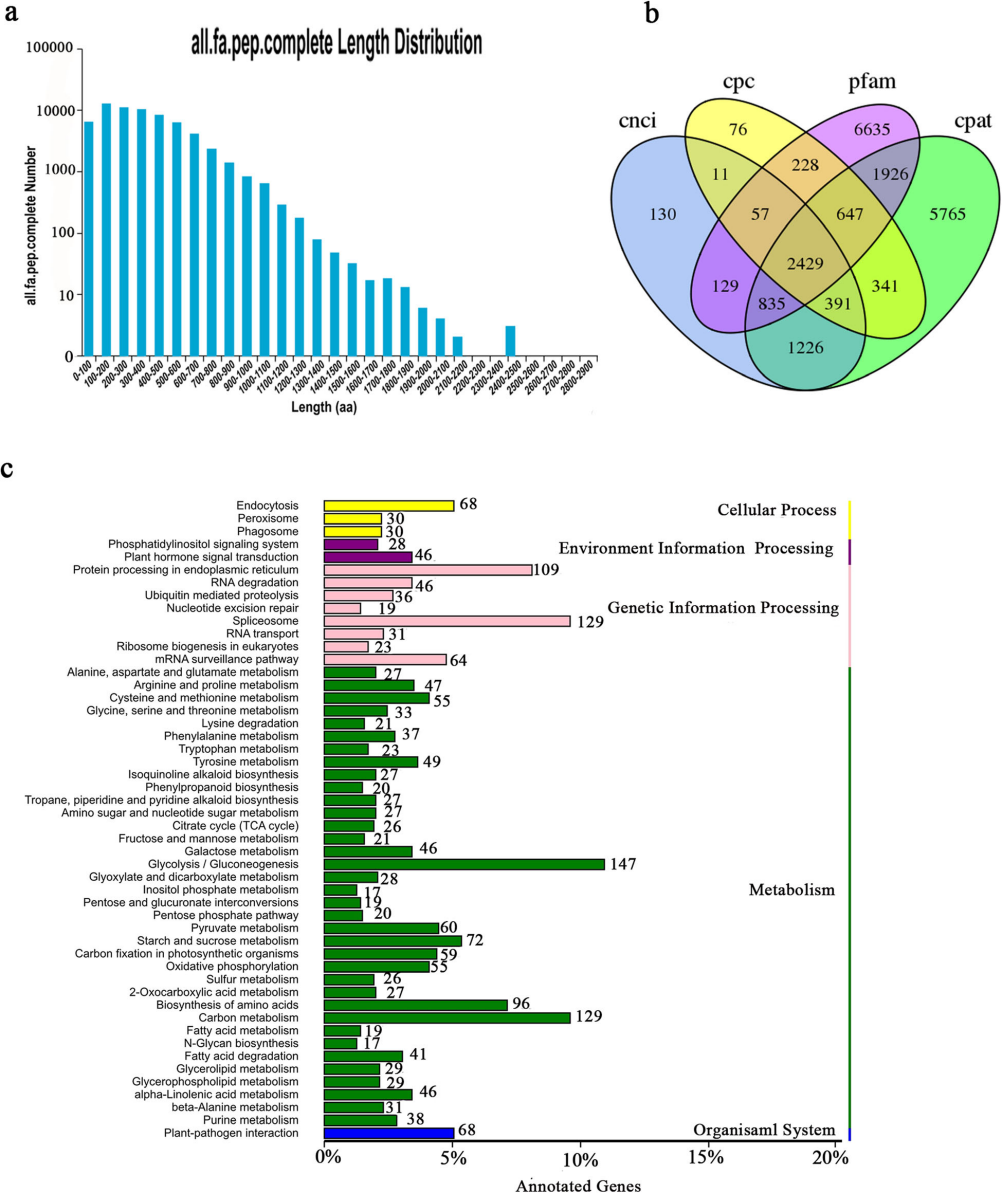
Prediction of CDS, lncRNAs and AS. **a** The distribution of CDS lengths with a complete open reading frame. **b** Venn diagram of the number lncRNAs predicted. **c** KEGG pathways of genes related to AS

To identify putative protein-coding sequences, we predicted 78,209 open reading frames (ORFs) using TransDecoder. In total, 65,227 coding sequences (CDSs) were identified with start and stop codons, and the distributions of the numbers and lengths of complete CDSs are shown in Fig. 1a. Among them, 12,630 transcripts were distributed in the 100–200-bp range.

A total of 79,424 sequences (167,351,883 bp) were examined, including 58,276 simple sequence repeats (SSRs) and 36,110 SSR-containing sequences (Additional file 2: Table S1). The number of sequences containing more than one SSR was 13,856, and the number of SSRs present in compound form was 10,041. In addition, most sequences are mononucleotides (33,533), dinucleotides (8610) and trinucleotides (14,026).

In this study, 2429 long noncoding RNAs (lncRNAs) were predicted by a coding potential calculator (CPC), the coding-non-coding index (CNCI), pfam protein structure domain analysis and the coding potential assessment tool (CPAT) (Fig. 1b), revealing candidate lncRNAs for future research.

A total of 6821 AS events were detected. Because no reference genome is available for caucasian clover, we could not identify the types of AS. Nonetheless, as AS is an important mechanism for regulating gene expression and producing proteome diversity, we show the results of these AS events in the KEGG enrichment (Fig. 1c), and the genes were found to be highly enriched in the following categories: “glycolysis/Gluconeogenesis” (147), “spliceosome” (129), “carbon metabolism” (129), “protein processing in endoplasmic reticulum” (109) and “biosynthesis of amino acids” (96).

### Transcript annotation

We annotated 77,927 (96.61%) transcripts in at least one of seven databases, including NCBI non-redundant protein (NR), Swiss-Prot (a manually annotated and reviewed protein sequence database), Gene Ontology (GO), Clusters of Orthologous Groups (COG), EuKaryotic Orthologous Groups (KOG), Protein family (Pfam) and Kyoto Encyclopedia of Genes and Genomes (KEGG). The number of detailed annotations for five of the databases (GO, COG, NR, KEGG and Swiss-Prot) is shown in a Venn diagram (Additional file 3: Figure S2).

**Fig. 2:**
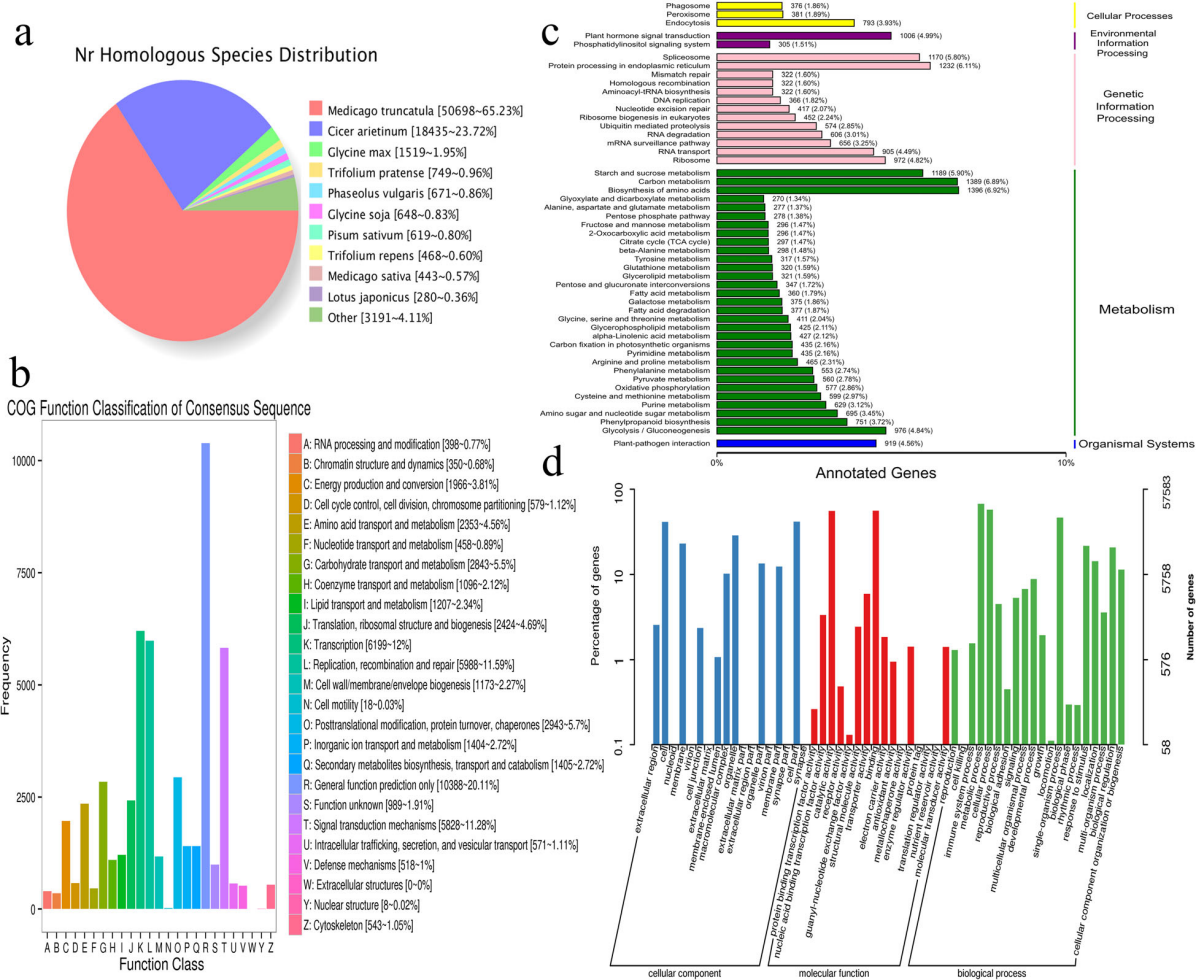
Transcripts annotated in four databases. **a** NR homologous species distribution diagram of transcripts. **b** COG function classification of transcripts. **c** KEGG pathway classification of transcripts. **d** Distribution of GO terms for all annotated transcripts

Through homologous species analysis comparing transcriptome sequences in the NR database, 77,721 transcripts were annotated. Approximately 65.23% (50,689) of the sequences were aligned to *Medicago truncatula* sequences, followed by *Cicer arietinum* (23.72%, 18,435) (Fig. 2a).

All the assembled transcripts were subjected to searches against the COG database to evaluate the effectiveness and completeness of the transcriptome annotation, and the results were divided into 26 main categories (Fig. 2b). The clusters “general function predicted only” (10,388), “transcription” (6199), and “replication, recombination and repair” (5988), represented three of the largest groups, followed by “signal transduction mechanisms” (5828) and “posttranslational modification, protein turnover, and chaperone” (2943).

A total of 33,383 (42.84%) transcripts were matched in the KEGG database and further classified into 128 KEGG pathways (Fig. 2c); “biosynthesis of amino acids” (1396), “carbon metabolism” (1389), “protein processing in endoplasmic reticulum” (1232), “starch and sucrose metabolism” (1189) and “spliceosome” (1170) were the most represented pathways.

Based on the GO analysis, 57,583 transcripts were enriched in the three ontologies (Fig. 2d) “biological process”, “molecular function” and “cellular component”. Transcripts involved in biological processes mainly included “metabolic process” (39,010), “cellular process” (33,383), and “single-organism process” (26,933). In molecular function, transcripts were mainly enriched in “binding” (32,350), “catalytic activity” (32,206), and “transporter activity” (3404). Regarding the “cellular component” category, the major classes of transcripts were related to “cell part” (24,076) “cell” (23,984) and “organelle” (16,648).

### Specifically expressed genes and statistics for DEGs

We investigated transcript expression levels in the five tissues, including taproot (T1), horizontal rhizome (T2), swelling of taproot (T3), rhizome bud (T4) and rhizome bud tip (T5) tissues, and T1 had the highest number of expressed genes (76,124), followed by T4 (75.978), T2 (75,885), T3 (74,396) and T2 (74,327) (Additional file 4: Figure S3a and Additional file 4: Figure S3b.). The number of genes co-expressed in each tissue was 68,241. Clean reads obtained by Illumina sequencing were compared with non-redundant transcripts to obtain position information on the transcripts and quantitative expression levels (Additional file 5: Table S2). Fragments per kilobase of transcript sequence per million base pairs sequenced (FPKM) values were used to represent the expression levels of genes. To determine specifically expressed genes in tissues and provide insight into the specialized developmental process, these genes (at least two repeats and FPKM> 0.1) with the top 5 average expression levels were selected for investigation (Table 2). Among them, FPKM values were higher in T2 and T5: F01_cb16574_c994/f1p0/1592 (mitogen-activated protein kinase, *MAPK3*) and F01_cb71 58_ c 94/f1p0/1000 (betaine aldehyde dehydrogenases, *BADH*).

**Table 2:** Specifically expressed genes of the top five FPKMs for each tissue

	Gene	FPKM	Description
T1	F01_cb14545_c33/f1p0/902	1.04	Diphosphoinositol-polyphosphate
	F01_cb17480_c9/f1p0/696	0.90	ADP-ribosylation factor 1
	F01_cb15376_c1/f1p0/1605	0.66	Protein of unknown function
	F01_cb14185_c1/f2p0/1231	0.50	Putative DNA-binding protein
	F01_cb8972_c16/f1p0/1728	0.42	Cytochrome P450
T2	F01_cb16574_c994/f1p0/1592	18.39	Mitogen-activated protein kinase
	F01_cb17761_c3939/f1p0/3262	1.57	SNF2 family N-terminal domain;
	F01_cb10989_c11/f1p0/1938	1.21	Cytochrome P450
	F01_cb16704_c9/f2p2/873	0.60	Plant invertase/pectin methylesterase inhibitor
	F01_cb8297_c8/f1p0/2003	0.56	EH-domain-containing protein;
T3	F01_cb8987_c27/f1p0/456	4.07	ADP-ribosylation factor 1-like
	F01_cb8782_c40/f44p1/1360	3.55	peroxidase
	F01_cb7280_c24/f1p1/2016	2.57	NAC domain-containing protein
	F01_cb7489_c3/f1p0/2140	2.10	PHD-finger
	F01_cb8820_c25/f1p0/1072	2.08	Ribosomal protein
T4	F01_cb17761_c89877/f1p0/2957	11.28	Auxin response factor
	F01_cb17761_c104162/f2p1/2284	11.02	BURP domain
	F01_cb16338_c23/f1p0/313	5.44	Calmodulin-like protein
	F01_cb17761_c21336/f1p0/3638	3.97	Leucine Rich Repeat
	F01_cb1066_c94/f1p0/2823	3.35	Probable galactinol—sucrose galactosyltransferase 2-like
T5	F01_cb7158_c94/f1p0/1000	146.47	Betaine aldehyde dehydrogenase 1
	F01_cb5102_c38/f1p0/684	38.70	Probable protein phosphatase
	F01_cb16574_c20256/f2p0/1154	36.04	60S ribosomal protein L2
	F01_cb9053_c18/f1p0/304	30.32	–
	F01_cb11585_c6/f2p0/314	20.12	–

**Fig. 3:**
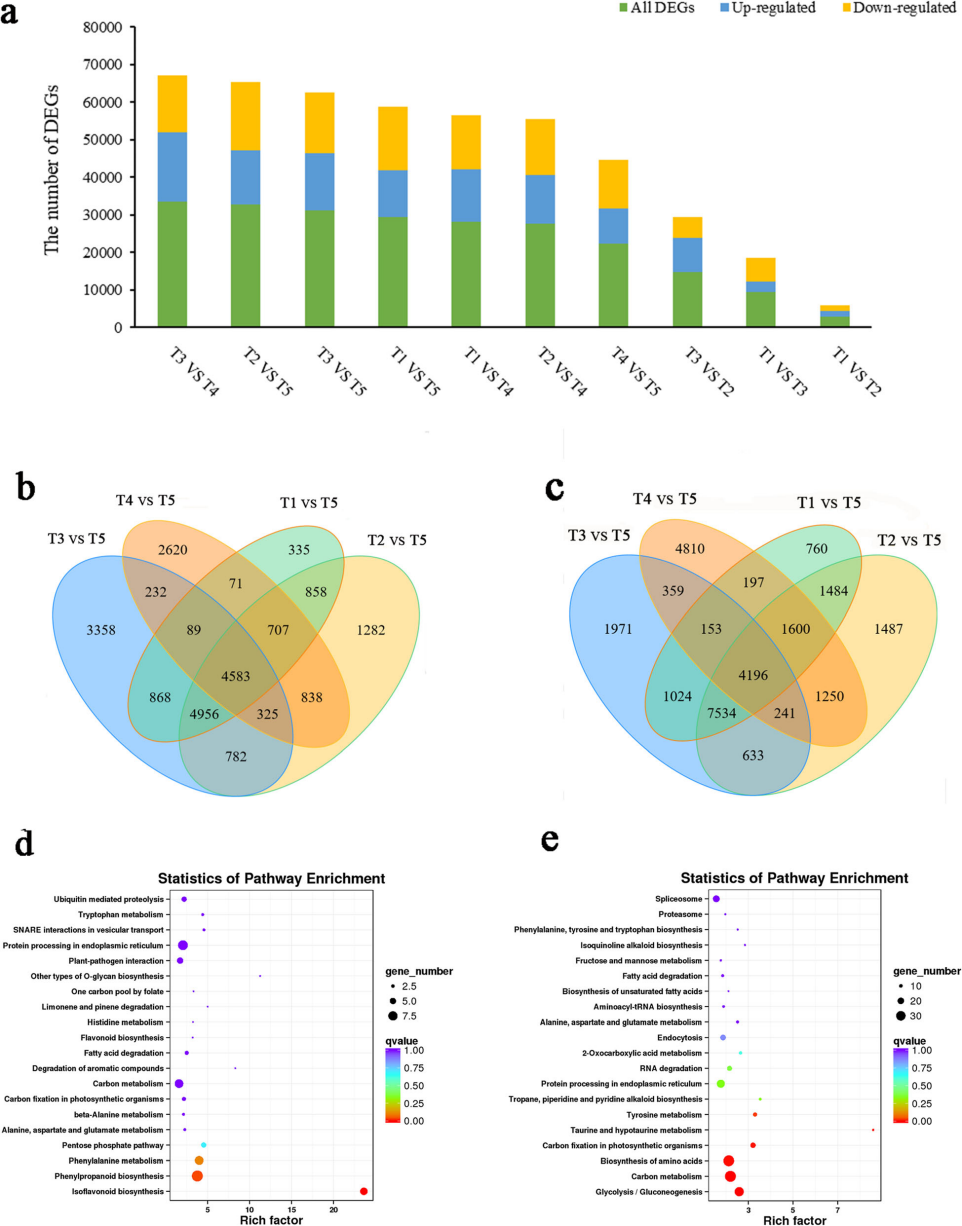
Differentially expressed genes statistics in the rhizome of caucasian clover. **a** The number of DEGs in different tissues. **b** Venn diagram showing upregulated genes in tissues compared to T5. **c** Venn diagram showing down-regulated genes in tissues compared T5. **d** Co-upregulated genes in KEGG enrichment for T5. **e** Co-downregulated genes in KEGG enrichment for T5

To identify gene expression differences in the development of the caucasian clover rhizome, we focused on identifying differentially expressed genes (DEGs) obtained by Illumina sequencing. As shown in the diagram of the DEGs distribution in different rhizome tissues (Fig. 3a), T3 and T4 had the largest number (33,612) of DEGs, with 18,372 up-regulated and 15,240 down-regulated genes. Moreover, T5 and other tissues (T1, T2, T3 and T4) had more DEGs; 4585 co-upregulated genes and 4196 co-downregulated genes were found in T5 compared with different tissues (Fig. 3b and c). With regard to the up-regulated genes in T5, only 65 were enriched in the KEGG analysis: “phenylpropanoid biosynthesis” (9), “protein processing in endoplasmic reticulum” (8), “phenylalanine metabolism” (7), “carbon metabolism” (7) and “isoflavonoid biosynthesis” (6) (Fig. 3d). For the down-regulated genes in T5, 231 genes were annotated, mainly involved “carbon metabolism” (35), “biosynthesis of amino acids” (34), “glycolysis/gluconeogenesis” (29), “protein processing in endoplasmic reticulum” (25) and “spliceosome” (21) (Fig. 3e).

### TFs prediction and WGCNA

**Fig. 4:**
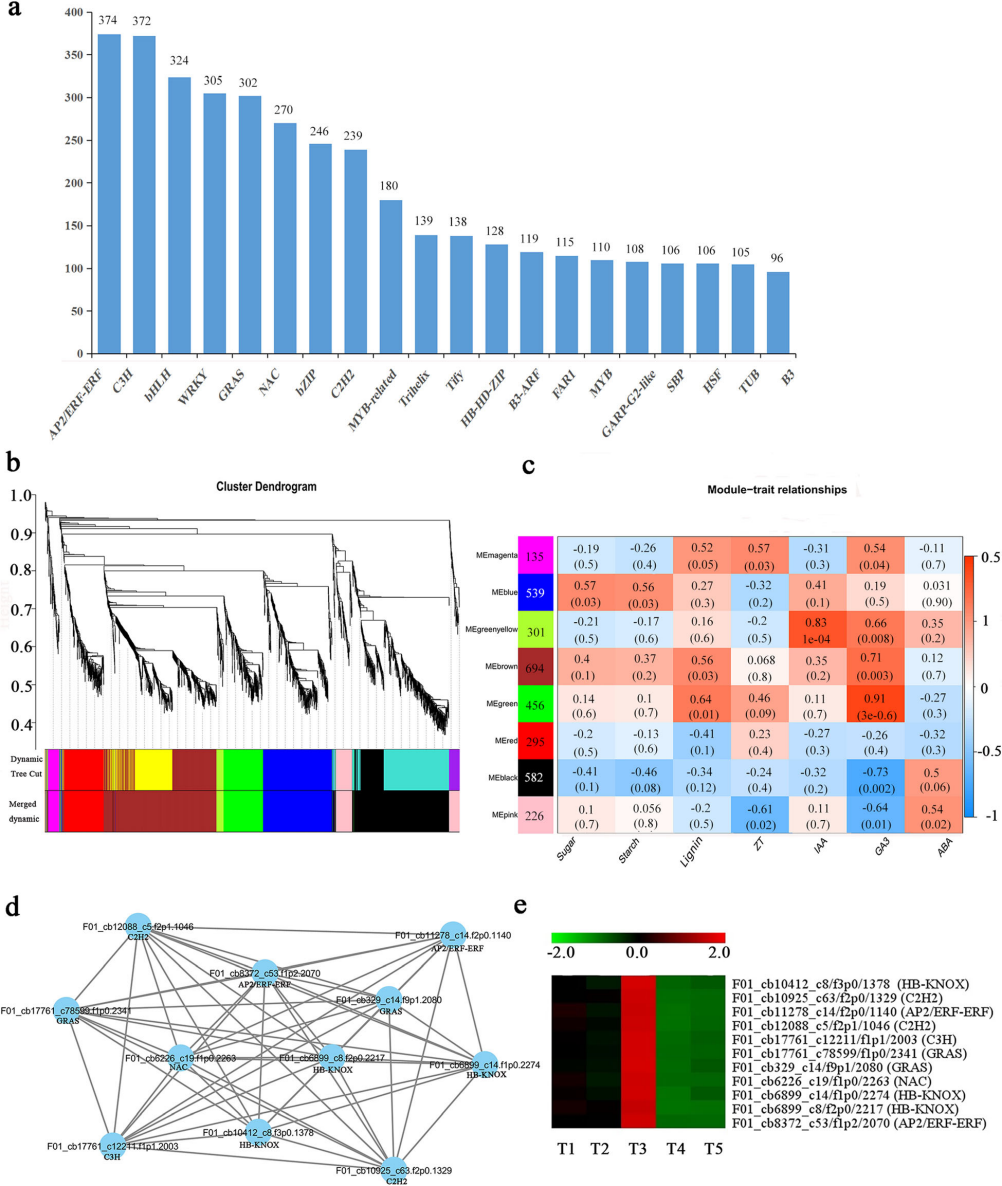
The results of TF WGCNA. **a** Number of top 20 TFs. **b** Hierarchical cluster tree showing co-expression modules identified by WGCNA. **c** Module-sample association relationships. **d** Correlation networks of hub genes in the green module. **e** Heatmap of hub genes in the green module

TFs play critical roles in plant growth and development. We examined 4501 putative TFs from 64 different families (Additional file 6: Table S3), and the top 20 TF families are shown in Fig. 4a. Most transcripts belonged to the AP2/ERF-ERF (374), C3H (372), bHLH (324), WRKY (305), GRAS (302), NAC (270), BZIP (246), C2H2 (239), and MYB-related (180) families. These TFs are widely involved in plant growth and responses to stress and are related to rhizome development.

We used weighted gene co-expression network analysis (WGCNA) to further explore the relationship between TFs (with filtering of TFs with an FPKM value < 1 and K-ME< 0.7) (Additional file 6: Table S3) and physiological characteristics in the rhizome of caucasian clover (Additional file 7: Table S4). Highly correlated TFs clusters are defined as modules, and TFs within the same cluster are highly correlated. WGCNA identified eight distinct modules (labelled with different colours in Fig. 4b). The correlation coefficients between the characteristic genes of each module of 10 different modules and each different sample (trait) are presented in Fig. 4c. Notably, the auxin (IAA)-trait and GA3-trait were significantly correlated with the MEgreenyellow modules and MEgreen modules (*r*^2^ > 0.8, *p* < 10^− 4^). Most genes of the green module were up-regulated for six traits, except for abscisic acid (ABA) and most of these TFs were mainly up-regulated in T3 (Additional file 8: Figure S5). We identified 11 hub genes based on the criteria of eigengene-based connectivity (kME) value≥0.99 and an edge weight value ≥0.5 in the green module based on the regulatory network (Fig. 4d). These hub genes mainly belong to the HB-KNOK, AP2/ERF-ERF, GRAS, C2H2, C3H and NAC families (MEgreen-GA3); moreover, these TF families were up-regulated in T1, T2 and T3, particularly in T3 (Fig. 4e) and may be related to the formation of nodules in the rhizome.

### Identification of hormone signalling-related genes in rhizome development

**Fig. 5:**
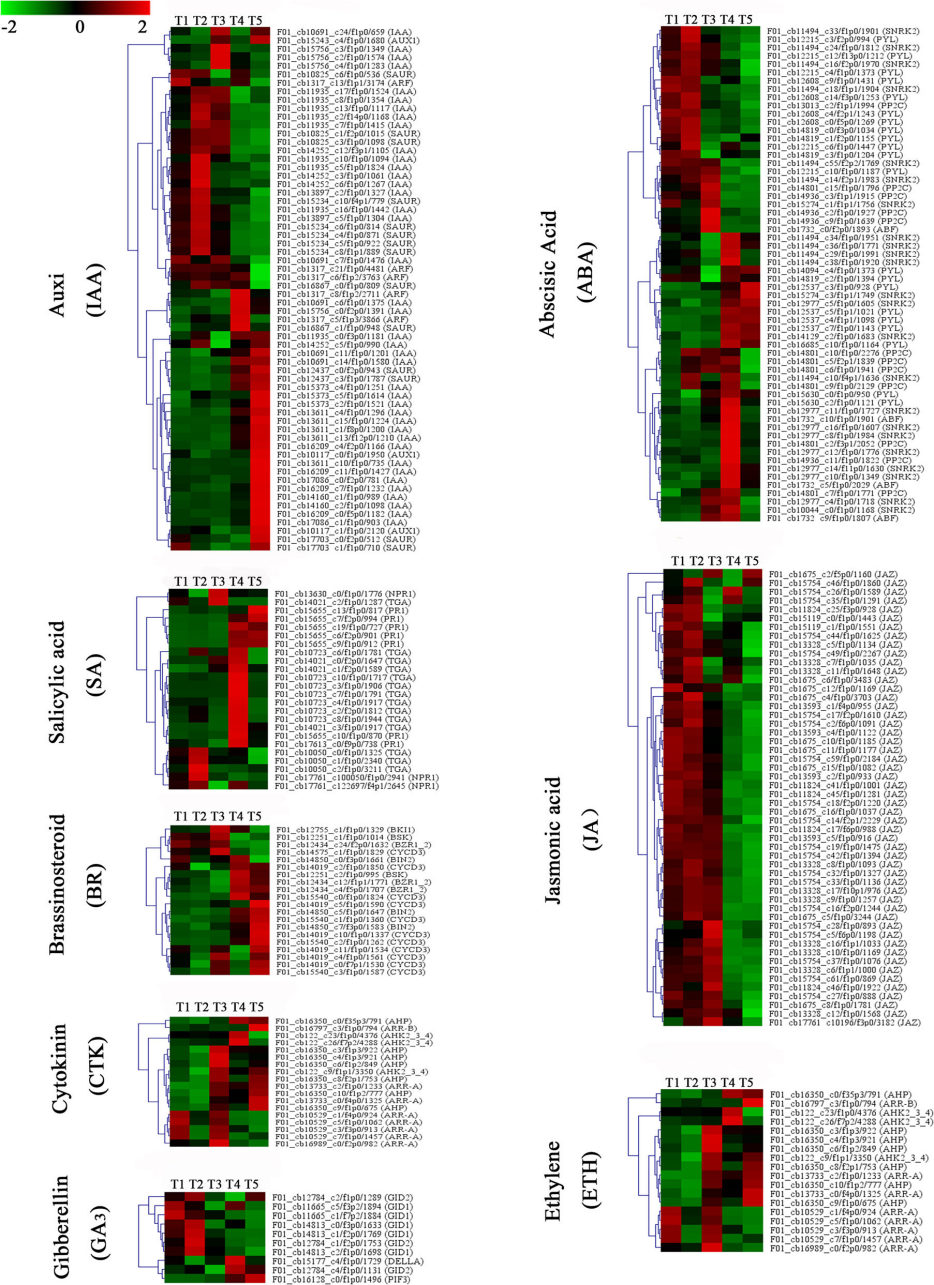
Heatmap of hormone signaling-related genes in the five tissues

Plant hormones play an important role in all aspects of development. Accordingly, we mapped DEGs to hormone signalling and transduction pathways for caucasian clover and analysed their expression in different tissues. In total, 276 DEGs are related to the synthesis and metabolism of eight hormones, including IAA, ABA, ethylene (ETH), cytokinin (CTK), gibberellic acid (GA), brassinosteroid (BR), jasmonic acid (JA) and salicylic acid (SA) (Fig. 5). The maximum number of genes (62) was found for IAA synthesis and metabolism, followed by ABA at 60, JA at 52, SA at 24, ETH at 28, BR at 20, CTK at 18 and GA at 10. All these significant genes related to hormones synthesis and metabolism exhibited different expression patterns in the different tissues. In addition, More genes were clearly up-regulated in T2 and T5 than other tissues. Regarding SA signalling, almost all genes belonged to the TGA family, with up-regulation only in T4. Most genes associated with BR signalling showed higher levels in T4 and T5. For CTK transduction, all crucial genes associated with the CTK signalling pathway were identified as DEGs. Only three genes showed no up-regulation trend in T3, which may be the tissues in which cells divide in large numbers. Only ten DEGs were associated with GA signalling; five genes were classified as *GID1* and significantly up-regulated in T2. Genes related to ABA signalling and transduction displayed no significant change. For JA signalling, 52 genes were annotated as JAZ, with up-regulation in T1, T2 and T3. In contrast, most ETH signalling genes exhibited higher expression in T3, and all were down-regulated in T2.

### Verification of gene expression by qRT-PCR

To confirm the accuracy of the genes obtained by RNA-seq, twelve genes, including six plant hormone signal transduction genes, three TFs and three genes belonging to other classes, were randomly selected for quantitative real-time RT-PCR (qRT-PCR) analysis (three replicates of each gene). Good reproducibility between the qRT-PCR and RNA-seq results was indicated by Pearson’s correlation analysis, thus verifying the accuracy and reliability of the RNA-seq data (Additional file 9: Table S5).

## Discussion

The rhizome of caucasian clover is unique among legume species, endowing this plant with particular clonal reproduction characteristics and resistance to stress. In this study, we obtained high-quality transcript sequences for the caucasian clover rhizome by PacBio and Illumina sequencing, and the results will contribute to our understanding of rhizome growth and development and provide a molecular foundation for further study.

AS is a vital mechanism regulating gene expression and producing protein diversity [42]. The numbers of AS events identified for the first time in the caucasian clover rhizome was found to be lower than that in *Medicago sativa* (7568) [43] but higher than that in *Trifolium pratense* (5492) [13]. Our study on the special characteristics of the caucasian clover rhizome was hindered by the lack of a reference genome, and the types of AS events are impossible to determine.

Many amino acids (phenylalanine, tyrosine and tryptophan) are not only important components of proteins but are also precursors of many secondary metabolites. These secondary metabolites are crucial for plant growth [44]. Similar to the rhizome of other plants (*Oryza longistaminata* and *Miscanthus lutarioriparius*) [12, 19], some basal metabolism plays an important role in the rhizome of caucasian clover, for example, carbon metabolism (1389) and starch and sucrose metabolism (1189)(Fig. 2c).

By analysing the specifically expressed genes in each tissue, we found that *MAPK3* (F01_cb16574_c994/f1p0 /1592) is mainly specifically expressed in T2 (Table 2). The MAPK family has been studied in tobacco, and it may be involved in growth, development, and responses to plant hormone and environmental signals [45]. T5 is very different from root tissues (T1, T2 and T3) (Fig. 3a), with more DEGs, and is the site of the formation of new aboveground parts. A specific gene with the highest FPKM in T5 was observed for *BADH* (F01_cb7158_c94/f1p0/1000) (Table 2). *BADH* genes are involved in glycine betaine synthesis and act as plant osmotic regulators, with important roles in abiotic stress [46]. Experiments have shown that *BADH c*an increase the tolerance of sweet potato and carrot against abiotic stresses such as salt stress, oxidative stress and low-temperature stress, thus maintaining cell membrane integrity [47, 48]. *BADH* was specifically expressed at a high level in T5, possibly because T5 is relatively more fragile than other tissues, and *BADH* may protect T5 to promot the growth of new plants. Thus, we speculate that defence and stress response play a vital role in the development of caucasian clover, which may be the reason for its ability to grow in extreme winter temperatures, or such responses may be a necessary condition for a large rhizome system.

TFs can offer insight into the gene-regulating networks controlling developmental programmes and are recognized as major contributors to a better understanding of root tissue differentiation and root development in response to internal growth regulators as well as environmental signals [49, 50]. The genes involved in hormone metabolism, cellulose synthesis energy, metabolism substance synthesis and transportation stress as well as expansion-related protein genes and TFs such as bHLH, TCP, WRKY, bZIP, MYB and NAC have been reported to in the formation of lotus rhizomes [24]. In addition, the AP2/ERF family accounts for the greatest number in the caucasian clover rhizome (Fig. 4a), and ethylene response factors, such as *BBM/PLT4* and *PLT1–3,* have been described as master regulators of root meristem initiation and maintenance in *Arabidopsis thaliana* [51, 52]. In *Raphanus sativus* and *Medicago sativa,* the abiotic stress response mechanism regulated by AP2/ERF has been carefully studied [53, 54]. The *Arabidopsis* NAC family member *NAC1* transduces IAA signals downstream of *TIR1* to promote lateral root development [55]. In the WRKY family, *WRKY75* was reported to be involved in regulating the nutrient starvation response and rhizome growth [56]. Notably we performed WGCNA clustering for TFs and found that 3 of 11 hub genes belong to the HB-KNOX family in MEgreen-GA3 (Fig. 4d). GA can suppress the effect of elevated KNOX protein expression, and modifying KNOX may alter the plant structure through local changes in GA levels [57]. In *Arabidopsis*, the *GA20ox1* mRNA level is reduced in leaves overexpressing the KNOX proteins STM or BREVIPEDICELLUS [56]. Moreover, in model plants, such as *Arabidopsis*, maize and tobacco, *KNOX* gene expression is confined to the shoot meristem and stem [58]. However, in the underground rhizome of caucasian clover, *KNOX* genes were identified as hub genes, especially in the main tissues of the swollen taproot (T3). Whether these finding are consistent with *KNOX* regulation of *Arabidopsis* meristems, stems and buds is worthy of further investigation [59]. Xi Cheng found that in pear plants co-expressing *KNOX* and *PbKNOX1,* these factors are involved in cell wall thickening and lignin biosynthesis, with inhibition of key structural genes involved in lignin synthesis [60].

Growing evidence indicates that hormones affect tillering growth and the formation of storage organs [12, 61]. Yi Kun’s research illustrated that 600 mg·L^- 1^ GA3 can promote the growth and development of caucasian clover rhizome and increase the contents of endogenous IAA, ZT and GA_3_ [62]. T1 and T2 showed high *GID1* expression and GA may be involved in photoperiod induction and regulation of the formation of storage organs and rhizome elongation [63]; therefore, TI and T2 may be key organs for nutrient storage. In addition, JAZ protein accumulated in roots (T1, T2 and T3). When pathogens invade and abiotic stress occurs, JAZ-MYC is believed to form an immune network, followed by JAZ protein degradation and MYC release [64]. T3 is closely linked to ETH and CTK-mediated pathways and may be responsible for root swelling. IAA has been demonstrated to activate root formation, while CTKs mediate root identity, early primordial disintegration and early loss of bud development initiation [65]. CTKs are conducive to rhizome enlargement but not to rhizome induction.

Genes related to the IAA anabolic pathway were downregulated in T3 and T4. IAA may not participate in root enlargement or induce bud production but may be closely related to lateral root development. T1, T2 and T3 might mainly function as storage organs, providing energy for plant growth. In addition, T4 may be relatively fragile and require the SA pathway to mediate immunity to prevent pathogen infection and to grow new plants. BR signalling is mainly involved in plant growth and plant morphology development, and related genes were upregulated in T5 [66]. T4 and T5 are mainly associated with resistance to stress and secondary metabolic pathways. Of course, hormones are not the only factors that regulate the development of apical meristems and lateral organs; they often cooperate with TFs to balance the maintenance of meristems and organogenesis.

## Conclusion

In summary, we provided a full-length transcriptome of the caucasian clover rhizome based on PacBio sequencing and Illumina sequencing, revealing gene expression patterns and annotation for different tissues. We highlighted the role of hormones and TFs in the rhizome of caucasian clover, investigated the expression of hormone pathway-related genes in different tissue of caucasian clover and identified 11 hub genes in TF and GA-related modules by WGCNA. In this study, a set of genes related to rhizome development was identified, laying the foundation for further functional genomics research on rhizome development.

## Methods

### Plant materials and RNA preparation

**Fig. 6:**
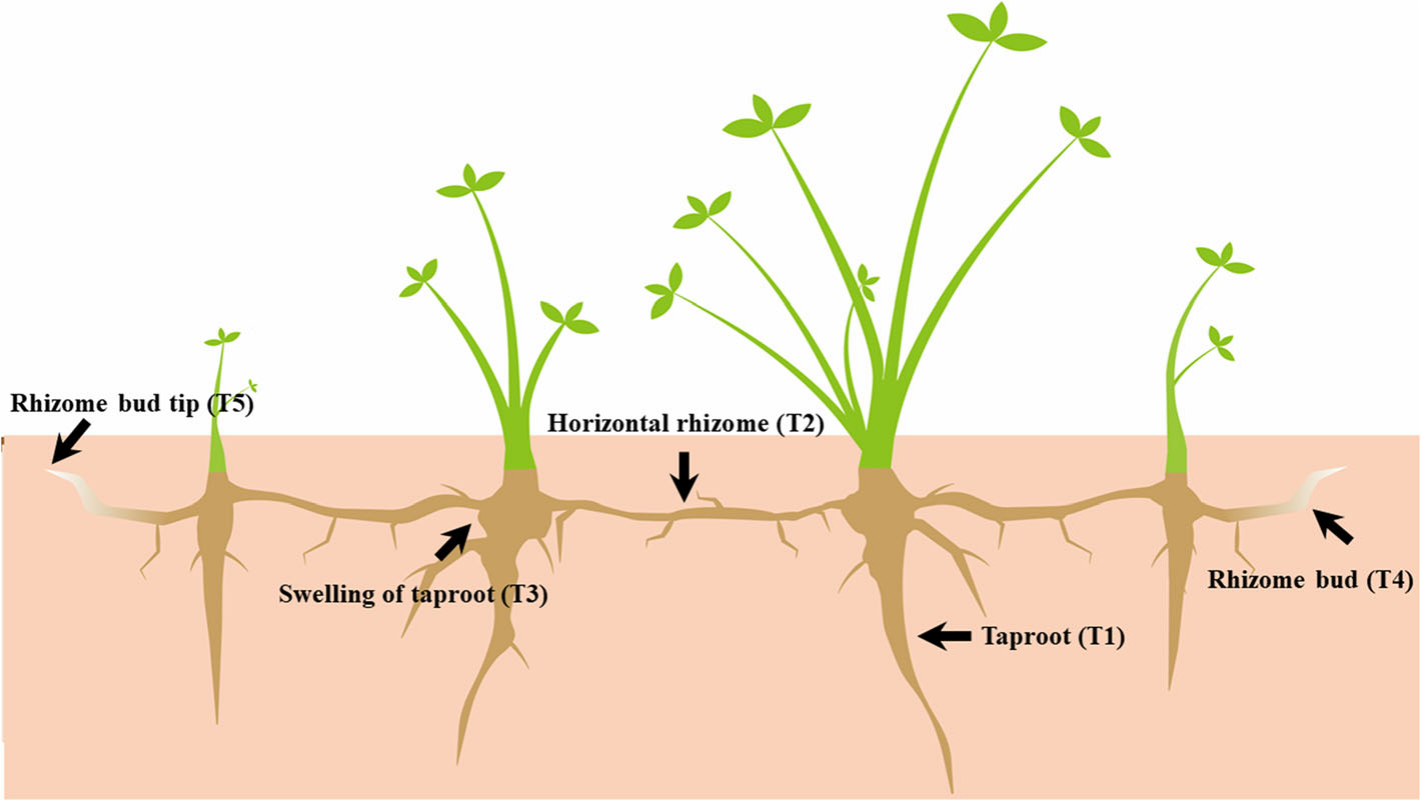
Schematic graph of tissues collected for PacBio sequencing and Illumina sequencing

The T1, T2, T3, T4 and T5 tissues of 3-year-old caucasian clover (Fig. 6) were collected from a test field at Northeast Agricultural University (E 126°14′; N 45°05′ in August 2018). We placed each sample collected into a 1.5-ml centrifuge tube with three replicates from five individual plants for each tissue to ensure RNA quantity. Each sample showed a good correlation (*R*^2^ > 0.8; Additional file 10: Figure S5). The original sources of the plant materials were introduced from Inner Mongolia Grass Variety Engineering Technology Research Center of Inner Mongolia Agricultural University. The Inner Mongolia Grass Variety Engineering Technology Research Center of Inner Mongolia Agricultural University undertook formal identification of the samples, provided details of the specimens deposited and allowed the collection. The IPNI Life Sciences Identifier (LSID) for caucasian clover is urn:lsid:ipni.org:names:522843–1. Plants were removed from the soil bed, and the roots were washed gently with running water, frozen in liquid nitrogen and immediately stored at − 80 °C. Total RNA was extracted using Trizol. RNA degradation and contamination were monitored by 1.2% agarose gel electrophoresis. The quantity and integrity of the extracted total RNA were determined using a NanoDrop and an Agilent 2100 bioanalyzer (Santa Clara, CA) [13]. For each RNA sample, 1 μg was pooled and sequenced by PacBio single-molecule long-read sequencing (PacBio Sequel, Menlo Park, USA) and Illumina sequencing (Illumina NovaSeq6000, California, U.S.A) in parallel.

### PacBio cDNA library preparation and sequencing

Full-length cDNA was synthesized using the SMARTerTM PCR cDNA Synthesis Kit and then subjected to full-length cDNA PCR amplification and repair of cDNA ends. The concentration and quality of the cDNA library were determined using the Qubit 2.0 Fluorometer and an Agilent 2100 bioanalyzer [67]. The 1–6-KB library was sequenced via PacBio Sequel.

### Illumina cDNA library construction and sequencing

First, 15 samples of eukaryotic mRNA were enriched with magnetic beads with oligo (dT) and randomly broken into small fragments in fragmentation buffer. First-strand cDNA was synthesized using six-base random hexamers with a small fragment of mRNA as a template. The cDNA was purified by AMPure XP beads after synthesis of second cDNA strand (adding buffer, dNTPs, RNase H and DNA polymerase I). Then, double-stranded cDNA was end repaired, adding a tail and the sequencing linker, and the fragment size was determined by AMPure XP beads [41]. The final cDNA library was assessed by PCR, and the quality of the cDNA library was determined using an Agilent 2100 bioanalyzer. The libraries were sequenced from both 5′ and 3′ ends using Illumina NovaSeq.

### PacBio sequencing data analysis

ROI sequencing was extracted using the Iso-seq pipeline with minFullPass = 0 and minPredictedAccuracy = 0.80. In ROIs, reads containing poly(A) tail signals and 5′- and 3′-cDNA primers are regarded as full-length non-chimera (FLNC) transcripts [68]. The consensus sequencing isoform was obtained by using the ICE algorithm. Finally, high-quality FL transcripts were classified with an accuracy greater than 99% using Quiver. CD-HIT (identity > 0.99) was used to remove redundant sequences in high-quality FL transcripts, and ultimately obtain non-redundant transcript sequences [69].

### Illumina sequencing data analysis

High-quality clean data (removing containing adapters, poly-N and low-quality reads from the raw data) were used for all downstream analyses [70]. These clean reads were then mapped to the PacBio reference genome sequence and normalized by converting the fragment counts to FPKM values. A differential expression analysis was performed using DESeq (v 1.10.1); a fold change ≥4 and an FDR < 0.01 based on DESeq were considered indicative of differential expression [71].

### Detection of SSR, ORFs, AS and lncRNA

SSRs in the transcriptome were identified using MISA (http://pgrc.ipk-gatersleben.de/misa/), and only transcripts ≥500 bp were detected.

TransDecoder software (https://github.com/TransDecoder/TransDecoder/releases) was employed to identify reliable potential CDSs from the transcript sequences based on the ORF length, log-likelihood score, and amino acid sequence comparison in the Pfam database.

We used Iso-SeqTM data directly to run all-vs-all BLAST with high-identity settings [72]. BLAST alignments that met all criteria were considered products of candidate AS events [73], with two HSPs (high segmentation pairs) > 1000 bp in the alignment. Two HSPs have the same forward/reverse orientation, and one sequence should be contiguous in the same alignment or have a small overlap of less than 5 bp. The other sequence should be different to show “AS gap”, and the contiguous sequence should align completely with the different sequences. The AS gap should be greater than 100 bp and at least 100 bp from the 3′/5′ end.

CPC [74], CNCI, CPAT [75] and Pfam [76] were used to distinguish nonprotein-coding RNAs from putative protein-coding RNAs. Transcripts with lengths greater than 200 nt and more than two exons were selected as lncRNA candidate genes.

### Functional annotation

Annotation information on the obtained nonredundant transcript sequences was based on BLAST in the following databases: NR, Pfam [76], KOG [77], COG, swiss-Prot [78], KEGG [79] and GO [80].

### Real-time RT- PCR

QRT-PCR was conducted in a 10-μl volume containing 0.5 μl of diluted cDNA, 0.2 μl of forward primer, 0.2 μl of reverse primer, and 1 × SYBR Premix Ex Taq II (TaKaRa) with the following conditions: 95 °C for 180 s, followed by 40 cycles of 95 °C for 15 s, 59 °C for 15 s and 72 °C for 15 s. The 2^-△△^Ct method was used to calculate relative expression levels [81]. All reactions were performed with three replicates. All primers used are shown in Additional file 11: Table S6.

## Supplementary information

**Additional file 1: Figure S1.** The output of ROI. **a** ROI read length distribution for 1-6 KB size bin. **b** ROI classification for 1-6 KB.

**Additional file 2: Table S1.** The prediction of SSRs.

**Additional file 3: Figure S2.** Venn diagram of the number NR, Swiss-prot, COG, GO and KEGG.

**Additional file 4: Figure S3.** The number of transcripts. **a** The number statistics of transcripts in five tissues. **b** Venn diagram of expressed transcripts among different tissues.

**Additional file 5: Table S2.** Statistics of comparison results between the Illumina sequencing data and the non-redundant transcripts

**Additional file 6: Table S3.** The prediction of caucasian clover rhizome transcription factor (TF) family.

**Additional file 7: Table S4.** The results of different physiological indicators. The bar chart of sugar, starch, lignin, ZT, GA_3_, IAA and ABA content.

**Additional file 8: Figure S4.** The heatmap of TFs in green module.

**Additional file 9: Table S5.** The results of qRT-PCR.

**Additional file 10: Figure S5.** Heatmap of the Pearson correlation coefficient of each sample.

**Additional file 11: Table S6.** Primers used for qRT-PCR.

## Data Availability

Raw reads of one combined PacBio library and one Illumina RNAseq library generated in this study are available from BioProject at NCBI (https://www.ncbi.nlm.nih.gov/bioproject/) under accession numbers PRJNA586585 and PRJNA588309, respectively.

## Abbreviations

OFRs: Open reading frames
CDSs: Coding sequences
SSRs: Simple sequence repeats
AS: Alternative splicing
LncRNA: Long non-coding RNA
TF: Transcription factors
KB: Kilobase
ROI: Reads of insert
ICE: ICE: Iterative clustering for error correction;
FL: Full-length
FLNC: Full-length non-chimeric
CNCI: Coding-Non-Coding Index
CPC: Coding potential
CPAT: Coding potential assessment tool calculator
FLNC: Full-length non chimera
COG: Clusters of Orthologous Groups
NR: NCBI non-redundant protein
GO: Gene Ontology
KOG: EuKaryotic Orthologous Groups
Pfam: Protein family
KEGG: Kyoto Encyclopedia of Genes and Genomes
DEG: Differentially expressed genes
FPKM: Fragments per kilobase of transcript sequence per million base pairs sequenced
WGCNA: Weighted gene co-expression network analysis
IAA: Auxin
ABA: Abscisic acid
ETH: Ethylene
CTK: Cytokinin
GA: Gibberellic acid
BR: Brassinosteroid
JA: Jasmonic acid
SA: Salicylic acid
BADHs: Betaine aldehyde dehydrogenases
MAPK: Mitogen-activated protein kinase
qRT-PCR: Quantitative real-time RT-PCR
