# Supplementary figures and images for: Revealing the full-length transcriptome of caucasian clover rhizome development
Source: BMC Plant Biol. 2020 Sep 16;20:429. doi: 10.1186/s12870-020-02637-4 (PMC7493993; doi:10.1186/s12870-020-02637-4)

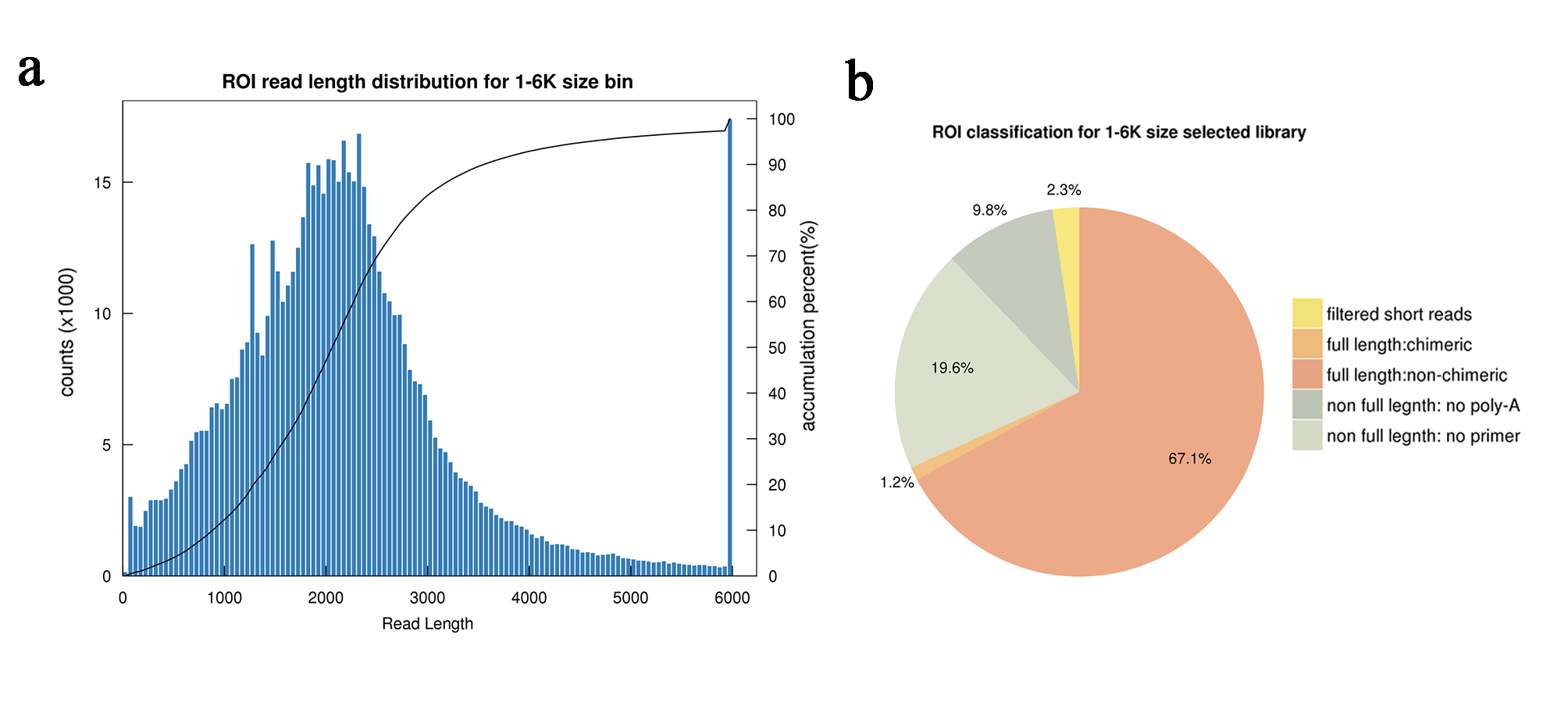

Supplement: Supplementary file 1 — Additional file 1: Figure S1. The output of ROI. a ROI read length distribution for 1-6 KB size bin. b ROI classification for 1-6 KB. [file 12870_2020_2637_MOESM1_ESM.tif]

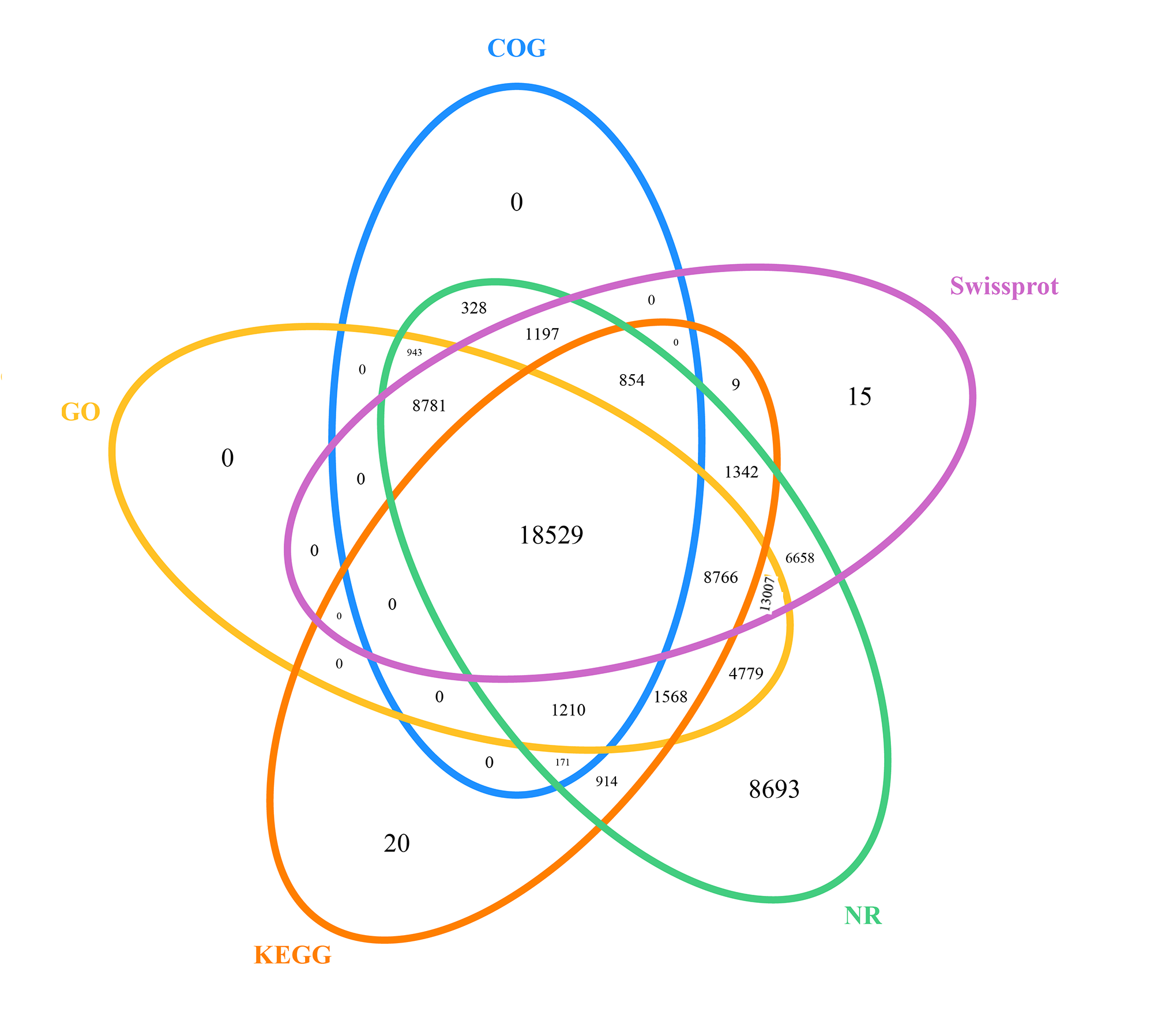

Supplement: Supplementary file 3 — Additional file 3: Figure S2. Venn diagram of the number NR, Swiss-prot, COG, GO and KEGG. [file 12870_2020_2637_MOESM3_ESM.tif]

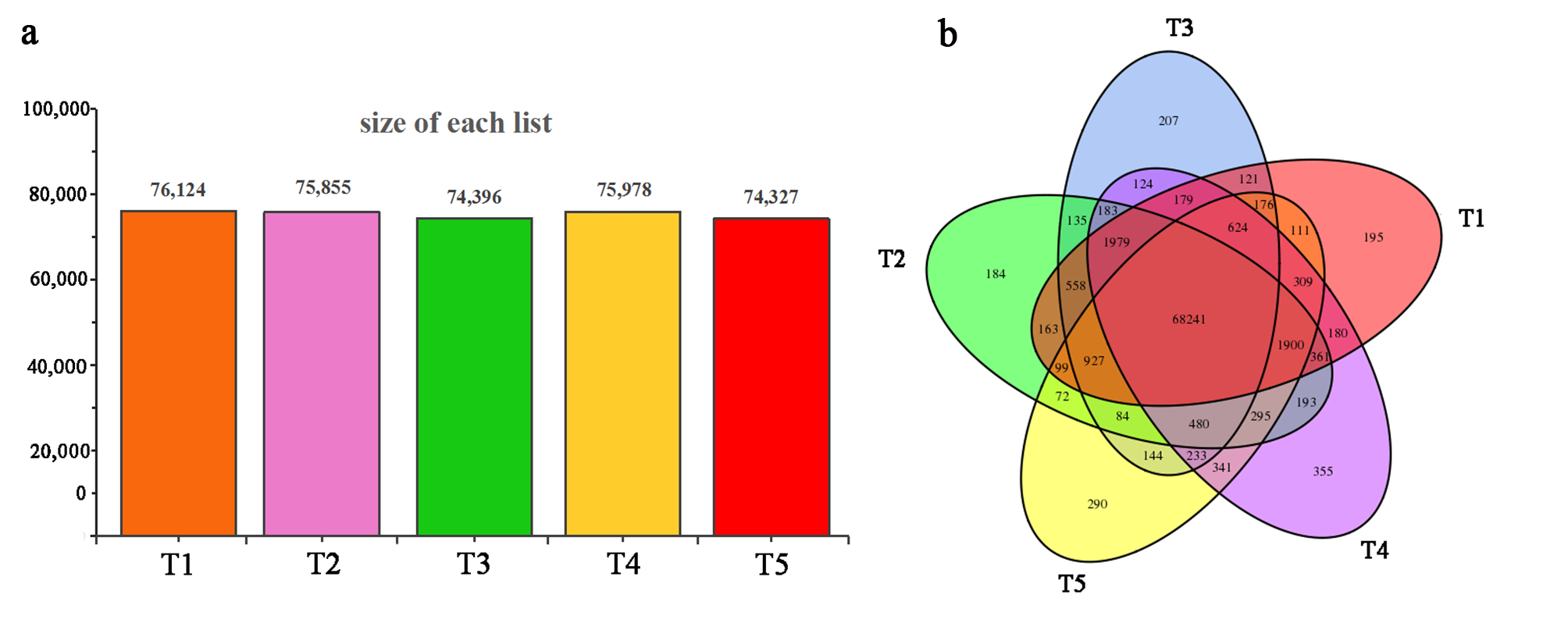

Supplement: Supplementary file 4 — Additional file 4: Figure S3. The number of transcripts. a The number statistics of transcripts in five tissues. b Venn diagram of expressed transcripts among different tissues. [file 12870_2020_2637_MOESM4_ESM.tif]

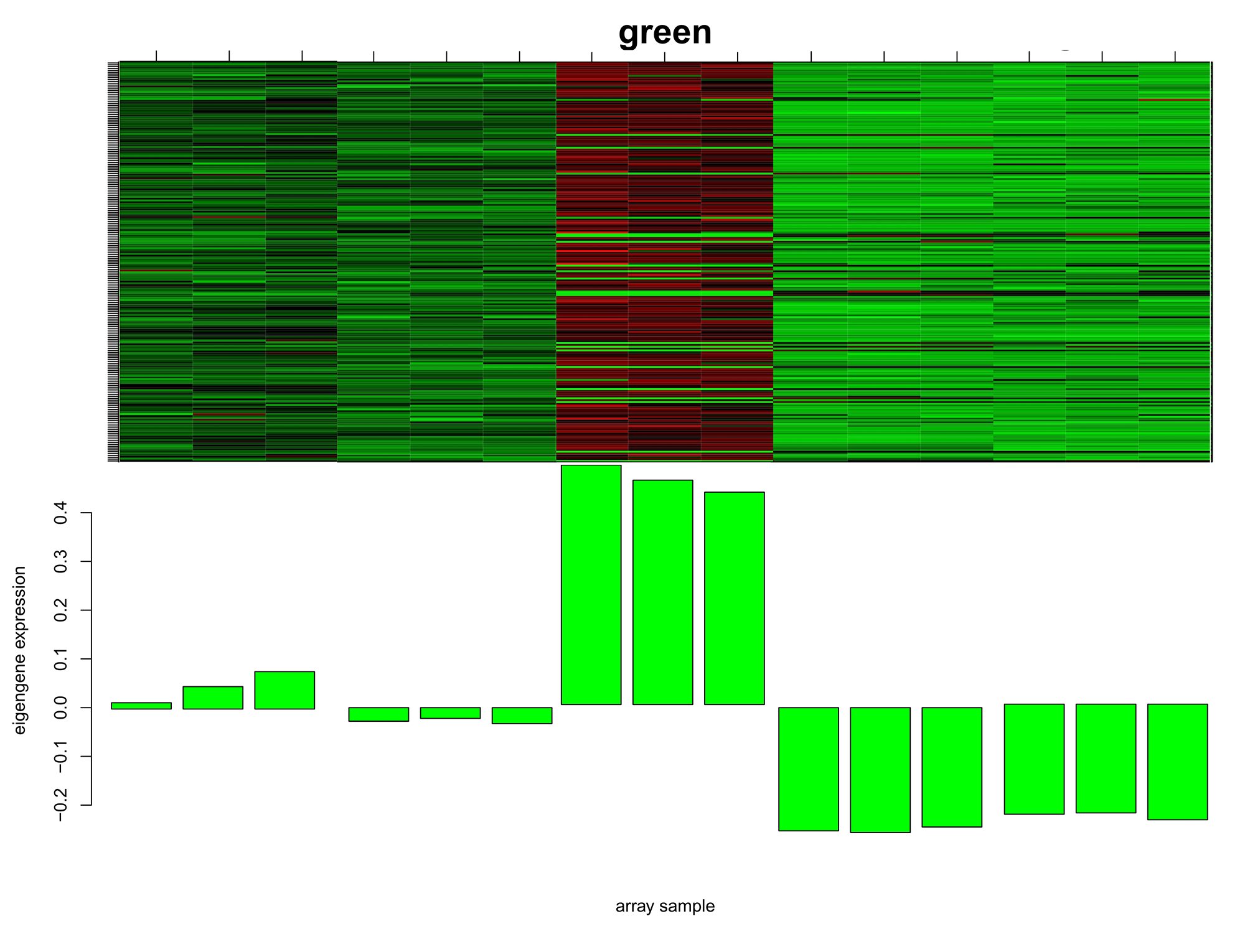

Supplement: Supplementary file 8 — Additional file 8: Figure S4. The heatmap of TFs in green module. [file 12870_2020_2637_MOESM8_ESM.tif]

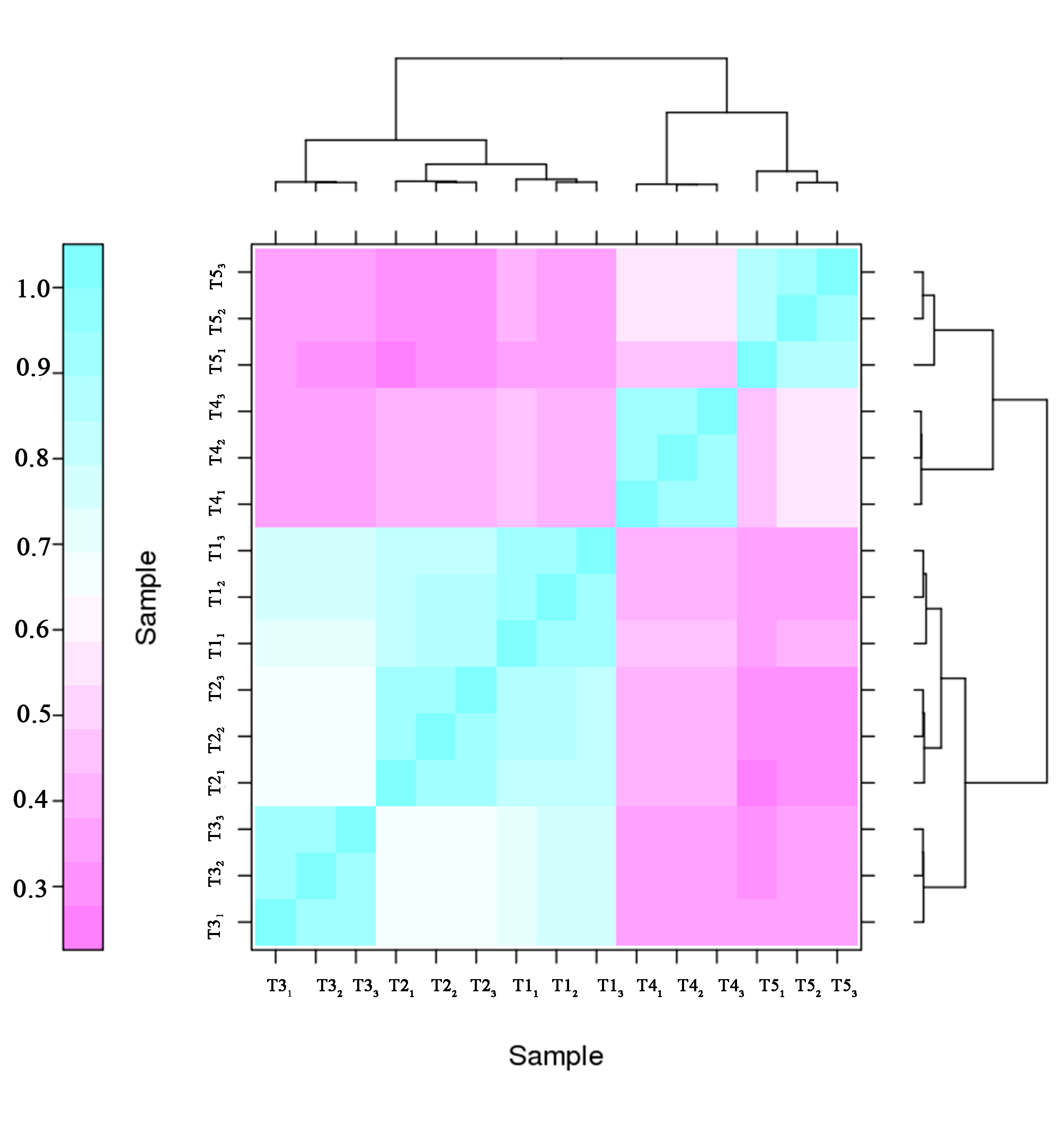

Supplement: Supplementary file 10 — Additional file 10: Figure S5. Heatmap of the Pearson correlation coefficient of each sample. [file 12870_2020_2637_MOESM10_ESM.tif]
